# Label-Free Electrochemical Aptasensor for Sensitive Detection of Malachite Green Based on Au Nanoparticle/Graphene Quantum Dots/Tungsten Disulfide Nanocomposites

**DOI:** 10.3390/nano9020229

**Published:** 2019-02-08

**Authors:** Qianqian Wang, Xiaofei Qin, Liping Geng, Yan Wang

**Affiliations:** Key Laboratory of Molecular and Nano Probes, Ministry of Education, Shandong Provincial Key Laboratory of Clean Production of Fine Chemicals, Collaborative Innovation Center of Functionalized Probes for Chemical Imaging in Universities of Shandong, College of Chemistry, Chemical Engineering and Materials Science, Shandong Normal University, Jinan 250014, China; sdnuwqq@163.com (Q.W.); sdnuqxf@163.com (X.Q.); sdnuglp@163.com (L.G.)

**Keywords:** electrochemical aptasensor, tungsten disulfide nanosheet, graphene quantum dots, Au nanoparticles, malachite green

## Abstract

A label-free electrochemical aptasensor was fabricated to sensitively determine malachite green (MG) based on Au nanoparticles/graphene quantum dots-tungsten disulfide nanosheet composite film modified glassy carbon electrode (AuNPs/GQDs-WS_2_/GCE). A facial strategy for the self-assembly of graphene quantum dots (GQDs) on tungsten disulfide nanosheets (WS_2_) was developed to fabricate 0D/2D nanocomposites. As-prepared GQDs-WS_2_ hybrids exhibited significantly enhanced electrocatalytic properties, and were first used as electroactive materials to construct electrochemical aptasensor. The AuNPs/GQDs-WS_2_/GCE was prepared through depositing Au nanoparticles on the surface of the GQDs-WS_2_ film, which acted as the electrochemical sensing matrix to covalently immobilize the aptamers of MG via the Au–S bond. In this label-free proposal, the aptasensor was applied to detect MG by monitoring voltammetric signal resulted from electrochemical oxidation of the MG captured by the aptamer. Under the optimized conditions, the aptasensor showed a wide linear range from 0.01 to 10 μM for MG detection with a low detection limit of 3.38 nM (S/N = 3). The method was applied to determination of MG in spiked fish samples and gave satisfactory results.

## 1. Introduction

Malachite green (MG) is a triphenylmethane dye which was widely applied in aquaculture because of its low cost and good antimicrobial activity [1]. Since 1990, many studies have showed that MG is highly toxic, mutagenic, and carcinogenic [2,3], which has been restricted or prohibited to be used as an antibacterial in fish farming due to potential hazards to human health resulting from its accumulation in aquatic products [4]. Thus, it is very significant to develop highly sensitive and selective methods to detect trace levels of MG residues in food safety assessment. Numerous methods have been reported for determination of MG, including enzyme-linked immunosorbent assay (ELISA) [5], a fluorometry method [6], liquid chromatography–mass spectrometry (LC-MS) [7], and surface-enhanced resonance Raman scattering (SERS) [8]. However, each of these methods has weakness, such as expensive instruments, long-time pretreatment processes and operational complexity. Electrochemical methods became alternative quantitative methods due to high sensitivity, low cost, and rapid response. Recently, electrochemical sensors have been reported in literature for the detection of MG [9,10,11,12], such as boron-doped diamond thin-film electrode [9], silica-modified electrode [10], carbon nanohorns modified electrode [11], and CeO_2_ nanoparticles modified glassy carbon electrode [12]. Nevertheless, these reported electrochemical methods still suffer from some undesirable limitation including low selectivity and limited sensitivity. Therefore, it is still highly desired to develop new protocols to improve sensitivity, selectivity and feasibility for detection of MG residues in aquatic food samples.

Aptamer based electrochemical biosensors, combing high sensitivity attributing to electrochemical methods with good specificity of aptamers, have received an increasing attention owning to their desirable advantages. In recent years, various kinds of electrochemical aptasensors have been successfully developed to be applied for the specific detection of various analytes, such as bisphenol A [13], potassium ion [14], cardiac troponin I [15], and adenocarcinoma gastric cancer cells [16]. However, most of the reported electrochemical aptasensors need labeling the aptamers or the targets with different redox probes to achieve highly sensitive detection. The labeling process not only has an effect on the binding affinity of aptamer toward target, but also results in operational complexity and high cost. In this work, we developed a new label-free electrochemical aptasensor based on nanocomposite film modified electrode for MG detection, which provided a facile, selective, sensitive, and fast voltammetric method for determining low concentrations of MG in fish samples. To the best of our knowledge, this is the first report in the literature for the amplified determination of MG based on label-free electrochemical RNA aptamer biosensor.

The electroactive materials play an important role in fabrication of electrochemical aptasensing platform due to their function of signal conversion and amplification. Among the various electroactive materials, good performance nanomaterials have been widely used because of excellent electrochemical properties such as high electronic conductivity and large specific surface area [17,18]. As graphene-analogous nanomaterials, transition metal chalcogenides (TMDs) have gained considerable investigation and application in the field of electronics and optics [19]. As two-dimensional (2D) layered nanomaterials, TMDs have a large specific surface area and are rich in active sites, which can obviously improve catalytic activity [20]. Recent studies have shown that the transition metal dichalcogenides, including MoS_2_ and WS_2_, possess extraordinary properties and potential applications in phototransistors, lithium batteries and electrocatalysts [21,22]. Compared with MoS_2_, WS_2_ nanosheets have higher intrinsic conductivity and better thermal and oxidative stability, indicating they are more suitable electroactive material for electrochemical sensor applications [23]. Furthermore, WS_2_ nanosheets have less toxic and better dispersion properties than graphene, implying that WS_2_ can be employed as novel nanomaterial for construction of biosensing interfaces [24]. To take advantage of WS_2_, scientists have tried to combine them with the other two-dimensional materials like reduced graphene oxide (rGO) to obtain better electronic conductivity. The formed heterostructures exhibit extraordinary properties superior to pristine WS_2_ nanosheets which have been applied in the hydrogen evolution reaction [25,26]. However, WS_2_/rGO nanohybrids prepared by the growth of WS_2_ nanosheets on graphene using the hydrothermal reaction have poor water dispersion, which limits their further application. As a rising star in graphene family, graphene quantum dots (GQDs) is zero-dimensional (0D) nanomaterial with only several nanometers diameter, which is composed of single- or few-layer graphene. Because of combination the nature properties of graphene and size-resulted quantum effects, GQDs show excellent electrochemical and photoelectrochemical properties [27,28]. However, to the best of our knowledge, the preparation and application of the GQDs-WS_2_ nanosheets hybrid in electrochemical sensor have never been reported in the literature. In this work, A new 0D/2D nanocomposite consisting of GQDs and WS_2_ nanosheets was prepared through a simple self-assembly technique. Held together by van der Waals forces, GQDs are uniformly dispersed on WS_2_ nanosheets to form heterostructures. The as-prepared GQDs-WS_2_ hybrids show enhanced electrochemical performance, which is attributed to the lower internal resistance through heterostructure formation of an interconnected conducting bridge for rapid electron transport between WS_2_ and GQDs, the wide range of WS_2_ oxidation states, and uniform distribution of the GQDs on WS_2_ nanosheets surface. Based on its excellent water dispersion property, large specific surface area, and significantly enhanced electrochemical properties, the GQDs-WS_2_ nanocomposite was first employed as electroactive material to modify glass carbon electrode which offered a new platform for fabrication of electrochemical aptasensor.

**Scheme 1:**
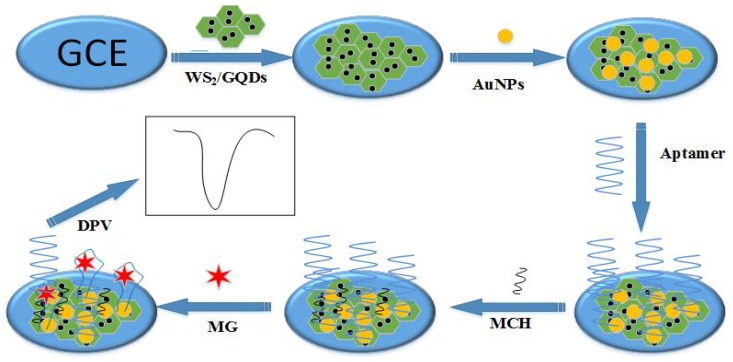
Fabrication process of the aptasensor for malachite green (MG) detection.

By combination of excellent electrocatalytic property of as-prepared nanomaterials and specific recognition reaction between aptamer and target, a novel electrochemical aptasensor was proposed for selective and sensitive determination of MG. In this label-free proposal, MG was specifically captured by aptamer immobilized on the sensor surface, next electrocatalytically oxidated by AuNPs/GQDs-WS_2_ to produce a sensitive voltammetric signal. As shown in Scheme 1, the AuNPs/GQDs-WS_2_/GCE was prepared through depositing Au nanoparticles on the surface of the GQDs-WS_2_ film, which acted as the electrochemical sensing matrix to covalently immobilize the thiolated aptamers of MG via Au–S bond. After MG was captured by the aptamer due to the specific recognition reaction, a well-defined anodic peak resulting from electrochemical oxidation of the MG on the modified electrode appears. The specific interaction between aptamer and MG may lead to MG analyte efficiently transferring from bulk solution to electrode surface, resulting in fast electron transfer between electroactive MG and aptasensor to produce strongly enhanced electrochemical signal. The oxidation peak current related to the MG was used as monitoring signal for determination of MG by differential pulse voltammetry. The experimental results demonstrated that the label-free electrochemical aptasensor showed good selectivity and high sensitivity in voltammetric measurement of MG, which provided a new approach for detection of MG residue at trace levels in aquatic food samples.

## 2. Materials and Methods

### 2.1. Chemicals

Graphene quantum dots (GQDs, 6–9 nm diameter, 0.8–1.2 nm thickness) and tungsten disulfide nanosheets (WS_2_, 20–500 nm diameter, 1–8 nm thickness) were obtained from Nanjing XFNANO Materials Tech Co. Ltd. (Nanjing, China). Malachite green, leuco malachite green (LMG), and 6-mercapto-1-hexanol (MCH) was purchased from Aladdin Reagent Co., Ltd. (Shanghai, China). Chloramphenicol, kanamycin, gentamicin sulfate, and methylene blue were received from Sinopharm Chemical Reagent Co., Ltd. (Shanghai, China). The thiolated MG binding aptamer was purchased from Shanghai Sangon Biotech Co. Ltd. (Shanghai, China), the base sequences are as follows 5’-SH-GGA UCC CGA CUG GCG AGA GCC AGG UAA CGA AUG GAU CC-3’. Tris-EDTA buffer (TE, 10 mM, pH 7.4) was used to prepare stock solution of aptamer. The phosphate buffer (PB, 0.067 M) at diverse pH was prepared by Na_2_HPO_4_ and KH_2_PO_4_.

### 2.2. Apparatus

A LK3200A electrochemical workstation (LANLIKE, Tianjin, China) was used for electrochemical measurements with a three-electrode cell, including a modified glassy carbon disk electrode (GCE, 3.0 mm diameter) as the working electrode, a platinum sheet electrode as the counter electrode, and a saturated calomel electrode as reference electrode. The surface morphology was characterized by high resolution transmission electron microscopy (HRTEM, JEM-2100, Tokyo, Japan) and field emission scanning electron microscopy (FE-SEM, S-4800, Hitachi, Tokyo, Japan). Electrochemical impedance spectroscopy (EIS) was carried out with a CHI660D electrochemical workstation (Chenhua Instrument Shanghai Co., Ltd., Shanghai, China).

### 2.3. Preparation of AuNPs/GQDs-WS_2_/GCE

The bare GCE was first polished with 0.05 μm α- alumina powder, and then ultrasonic washed with doubly distilled water for 10 min before its modification. The self-assembled GQDs-WS_2_ nanocomposites were fabricated as follows; 1.0 mg WS_2_ and 1.0 mg GQDs were dispersed in 2.0 mL doubly distilled water by sonication for 60 min to obtain a stable black suspension. Then a 5-μL aliquot of GQDs-WS_2_ nanocomposite was dropped onto the surface of clean GCE, which was dried at room temperature. The obtained electrode was noted as GQDs-WS_2_/GCE. For the sake of comparison, WS_2_/GCE was prepared by the similar procedures under the same conditions. 

A trisodium citrate reduction method was used to prepare AuNPs according to the previous report with a slight modification [29]. Briefly, under vigorous stirring, 0.4 mL of 388 mM trisodium citrate solution was rapidly added into 50 mL of 254 μM boiling HAuCl_4_ solution. After the color of solution changed from yellowish to wine red, it was continually heated for 15 min under reflux. Finally, the synthesized AuNPs solution was slowly cooled to room temperature under stirring. After filtration through 0.8-μm microporous membrane, the obtained AuNPs solution was stored at 4 °C. After 5 μL of AuNPs was modified on GQDs-WS_2_/GCE through physical adsorption, the prepared electrode was dried at room temperature, which was taken as AuNPs/GQDs-WS_2_/GCE.

### 2.4. Preparation of Electrochemical Aptasensor

The aptamer was immobilized on AuNPs/GQDs-WS_2_/GCE via Au–S bond between AuNPs and thiolated aptamer. The electrode was incubated with 5 μL of 5.0 μM aptamer at 4 °C overnight. After that, unbound aptamers were removed from electrode surface by rinsing with Tris-EDTA buffer. The obtained electrode was taken as aptamer/AuNPs/GQDs-WS_2_/GCE. Subsequently, 5 μL of 1 mM MCH solution was dropped on aptamer/AuNPs/GQDs-WS_2_/GCE for 60 min to block nonspecific sites. After being washed with pH 7.0 PB thoroughly, the as-prepared MCH/aptamer/AuNPs/GQDs-WS_2_/GCE was incubated in various concentrations of MG for 2 h at room temperature. Finally, the MG/MCH/aptamer/AuNPs/GQDs-WS_2_/GCE was washed with PB, which was applied to electrochemical measurement (Scheme 1).

### 2.5. Experimental Methods

After incubation of MG, electrochemical detection was performed immediately. Differential pulse voltammetric (DPV) and cyclic voltammetric (CV) measurements were employed with three electrodes in PB. The DPV experiments were carried out at a sweep potential from +0.2 V to +0.8 V with pulse amplitude of 30 mV. The cyclic voltammograms were recorded in the range from 0.0 V to +0.8 V with 100mVs^−1^ scan rate. All experiments were performed at room temperature.

### 2.6. Sample Preparation

The fish purchased from a local supermarket was used as MG-free sample. After the fish skin and bones were removed, fish meat was crushed and homogenized by blender. According to the protocol reported previously, the extraction method used is as follows [30]. An accurately weighed 5.0 g of homogenized fish meat samples were spiked with appropriate amounts of MG. After 9 mL of methanol was added into the spiked sample, the mixture was ultrasonic extracted for 15 min. Then it was centrifuged at 2500 g for 10 min. Finally, the collected supernatant was filtrated by 0.45-μm nylon membrane for future use.

## 3. Results and Discussion

### 3.1. Characterization of AuNPs/GQDs-WS_2_ Nanocomposites

**Figure 1:**
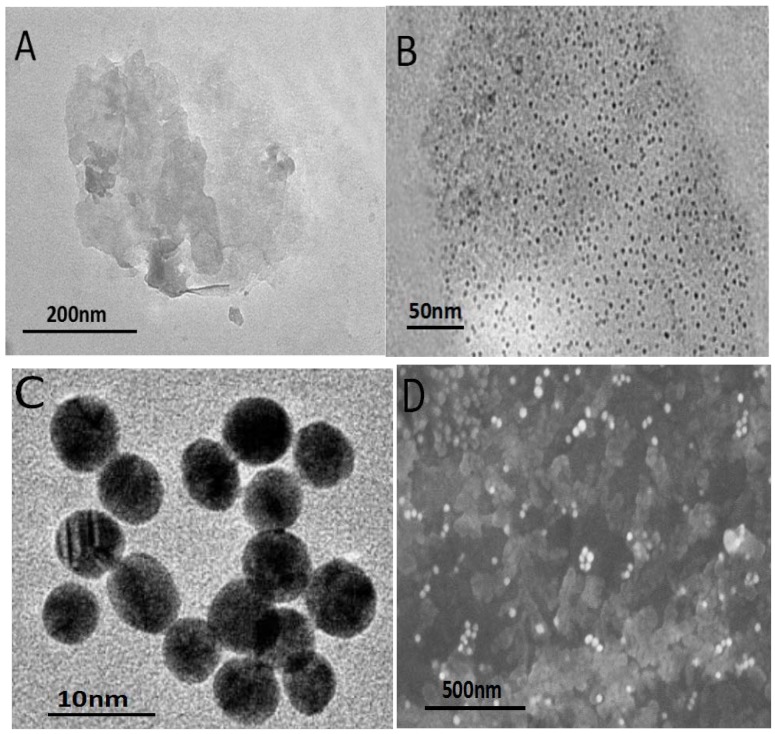
(**A**) TEM image of a tungsten disulfide (WS_2_) nanosheet. (**B**) TEM image of graphene quantum dots-tungsten disulfide nanosheet composite film modified glassy carbon electrode (GQDs-WS_2_). (**C**) TEM image of gold nanoparticles (AuNPs). (**D**) SEM image of AuNPs/GQDs-WS_2_ nanocomposite.

The morphological character of synthesized nanomaterials was investigated by SEM and TEM. Shown in Figure 1A,B is the TEM images of WS_2_ nanosheets and GQDs-WS_2_, respectively. In Figure 1A, the two-dimensional thin-layer WS_2_ nanosheets were observed, demonstrating that the samples were composed of graphene-like few-layer nanosheets with lateral dimensions varied between 20 and 500 nm. The TEM of GQDs-WS_2_ (Figure 1B) showed that GQDs with average diameter of ~8 nm are uniformly distributed on the WS_2_ nanosheets to form 0D/2D hybrid nanostructures via the van der Waals interactions between GQDs and WS_2_ nanosheets [31]. Furthermore, the usual aggregation and stacking between individual WS_2_ nanosheet were significantly inhibited in the GQDs-WS_2_ heterostructure due to the introduction of GQDs with wonderful dispersing performance, which can improve the water dispersion property of the hybrid and enhance the specific surface area. The TEM image of as-prepared AuNPs is presented in Figure 1C, demonstrating that AuNPs was homogeneous spherical particle with average diameter of 13 nm, which exhibited good monodispersity and stable solubility. The SEM image of AuNPs/GQDs-WS_2_ nanocomposite shown in Figure 1D clearly revealed that the obtained nanocomposite consisted of a large number of foil-like thin sheets, and AuNPs were well distributed on GQDs-WS_2_ flakes. Due to the synergy effects of three nanomaterials, the nanohybrids possess increased effective surface area, more activity sites and better conductivity.

**Figure 2:**
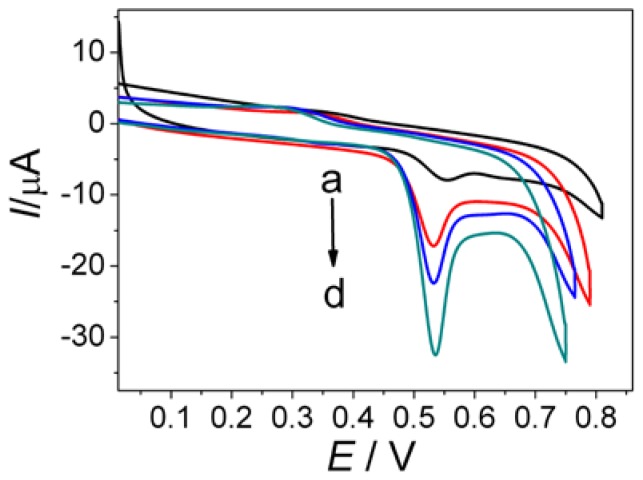
Cyclic voltammetry responses of the bare glassy carbon electrode (GCE) (**a**), WS_2_/GCE (**b**), GQDs-WS_2_/GCE (c), and AuNPs/GQDs-WS_2_/GCE (**d**) towards 0.5 mM MG in pH 7.4 phosphate buffer. Scan rate: 100 mV s^−1^.

To investigate the electrochemical properties of AuNPs/GQDs-WS_2_ nanocomposites, the electrochemical response to MG at different modified electrode was studied by cyclic voltammetry (Figure 2). As shown in curve a, a weak oxidation peak appeared at approximately +0.59 V using the bare GCE, indicating an irreversible redox behavior of MG and a slow electron transfer kinetic at unmodified electrode. After modification of WS_2_ nanosheets on the bare GCE, the oxidation peak current of MG significantly increased, as well as oxidation peak potential obviously negative shifting (curve b). This result demonstrated that WS_2_ film had a great electrochemical activity, which can enhance oxidation reaction of MG by electrocatalysis. In contrast, the GQDs-WS_2_/GCE (curve c) showed 4-fold increase in oxidation peak current than curve a, suggesting that GQDs in the hybrid film had the ability to enhance current responses of MG. In great contrast, the largest oxidation peak located at +0.53V was observed at the AuNPs/GQDs-WS_2_/GCE (curve d), which is approximately six times larger than that at the bare GCE. Moreover, the oxidation peak potential underwent negative shifts at AuNPs/GQDs-WS_2_/GCE by comparison with bare GCE. These phenomena clearly indicate that AuNPs/GQDs-WS_2_ composite film possess outstanding electrocatalytic activity towards the oxidation of MG though accelerating the rate of electron transfer and lowering overpotential. Based on the above results, it can be concluded that the synergistic catalysis effect of AuNPs and GQDs-WS_2_ hybrids can remarkably boost electrochemical oxidation of MG on electrode surface to improve detection sensitivity.

### 3.2. Electrochemical Performance of Aptamer Sensor

**Figure 3:**
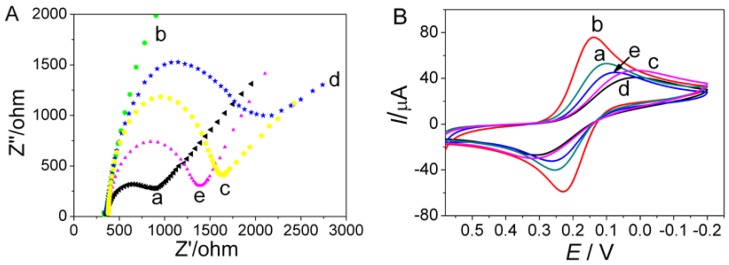
(**A**) Electrochemical impedance spectroscopy (EIS) spectra of the designed aptasensor at different modification stage. (**B**) CV responses of different modified electrode. (**a**) bare GCE, (**b**) AuNPs/GQDs-WS_2_/GCE, (**c**) aptamer/AuNPs/GQDs-WS_2_/GCE, (**d**) MCH/aptamer/AuNPs/GQDs-WS_2_/GCE, and (**e**) MG/MCH/aptamer/AuNPs/GQDs-WS_2_/GCE. The CV measurements were performed in 0.1M KCl solution containing 5 mM K_3_ [Fe(CN)_6_] at a scan rate of 50 mV s^−1^.

Electrochemical impedance spectroscopy (EIS) was used to study the electrochemical behavior of the aptasensor and monitor its preparation process. Figure 3A depicted Nyquist plots obtained at different steps of aptasensor preparation using [Fe(CN)_6_]^3−^/^4−^ as redox probe, in which the linear portion at lower frequency region corresponds to diffusion limited process and the diameter of semicircle at higher frequency region equals to electron transfer resistance (*R_et_*) [28]. Compared with bare GCE (curve a), the resistance *R_et_* obviously decreased after modified with AuNPs/GQDs-WS_2_.The impedance plot for AuNPs/GQDs-WS_2_/GCE (curve b) showed a near straight line, indicating that electron transfer of the redox probe was greatly accelerated due to the excellent charge transport efficiency of modified nanomaterials. The magnified Nyquist plot of AuNPs/GQDs-WS_2_/GCE near the origin is given in Figure S1, in which a small semicircle at very high frequencies was observed, indicating a low charge-transfer resistance. Moreover, it can be found that the low frequency straight line has a slope much greater than 1, indicating an ideal capacitance behavior and better electronic conductivity of AuNPs/GQDs-WS_2_ ternary nanocomposites [32]. After immobilizing the aptamer on the surface of AuNPs/GQDs-WS_2_/GCE, a drastically increased *R_et_* value was observed (curve c), implying successful assembly of aptamers of MG on the electrode surface. This increase in resistance is due to the electrostatic repulsion between the negatively charged redox probe and phosphate backbone of the aptamer [33]. Subsequently, the *R_et_* value significantly increased in curve d because the immobilized MCH hindered interfacial electron transfer. After the aptasensor was incubated with MG, the semicircle diameter markedly decreased (curve e). This appearance might be aroused by the conformational change of the aptamer induced by analyte. The forming of aptamer–MG bioaffinity complexes can induce conformational change of aptamer, which made the reception of the redox probe to the aptasensor surface more freely, resulting in decrease in electrode resistance [34]. The above EIS results indicated that the aptasensor had been successfully fabricated, which could be applied for detection of MG.

The electrochemical performance of aptasensor at different stages during its fabrication process was further evaluated by CV measurements, which were performed in 5.0 mM K_3_Fe(CN)_6_ solution to test electron transfer efficiency of different electrodes. It can be seen in Figure 3B curve a, bare GCE showed a pair of typical redox peak of K_3_Fe(CN)_6_ with Δ*E*_p_ as 0.12V (Δ*E*_p_ = *E*_pa_ − *E*_pc_). After surface modification with the AuNPs/GQDs-WS_2_ nanocomposite, a significantly increased CV signal with decrease in the peak potential separation (Δ*E*_p_ = 0.07 V) was obtained in curve b. This was attributed to the synergistic effect from electroactive GQDs, WS_2_, and AuNPs, which can increase the effective electrode surface area and improve electron transfer efficiency. Next, the peak current gradually decreased with the aptamer anchoring and MCH blocking (curve c and d). This phenomenon could be ascribed to the increased steric hindrance effect of the modified oligonucleotides and MCH molecules which gradually blocked the electron transfer of electrochemical probes on GCE interface. When the MCH/aptamer/AuNPs/GQDs-WS_2_/GCE incubated with MG solution, peak current mildly increased along with decreased Δ*E*_p_ (curve e), which was attributed to specific interaction between aptamer and MG. This result implied that conformational change of aptamer induced by aptamer–MG bioaffinity complexes can bring the electroactive MG molecules close to the electrode surface, which promotes the electron transfer of the redox probe on the GCE interface [34]. The above current change tendency was well in accordance with that of EIS spectrum, further demonstrating that the AuNPs/GQDs-WS_2_ nanocomposites have excellent electrocatalytic property.

### 3.3. Electrochemical Aptasensing of MG

**Figure 4:**
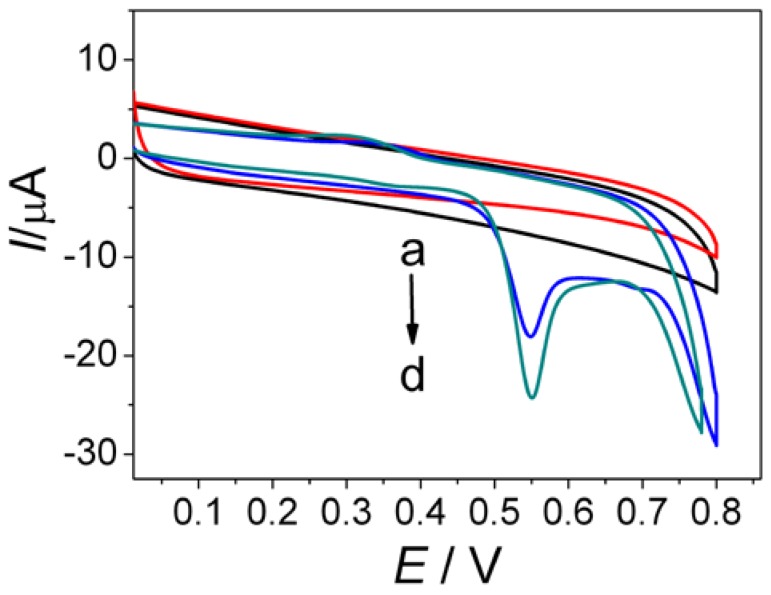
CV responses of the aptasensor toward blank control (**a**), 1.0 μM MG (**c**), and 10 μM MG (d). Curve b shows the CV response of the AuNPs/GQDs-WS_2_/GCE toward 1.0 μM MG in pH 7.4 phosphate buffer. Scan rate: 100 mV s^−1^.

In this label-free aptasensing proposal, MG was first captured onto the aptasensor surface by the aptamer recognition reaction, which was then electrocatalytically oxidated to produce a sensitive electrochemical signal for quantitative analysis. CV was used to study this signal transduction mechanism by measuring electrochemical response of aptasensor towards MG. As can be seen in Figure 4, CV recorded for the aptasensor (MCH/aptamer/AuNPs/GQDs-WS_2_/GCE) in pH 7.4 phosphate buffer did not display any redox peak (curve a). While the aptasensor was incubated with 1.0 μM MG, an obvious oxidation peak was observed at +0.53V in the CV (curve c), which was well in accordance with the electrochemical behavior of MG at AuNPs/GQDs-WS_2_/GCE. The result was also similar to the observation previously reported in the literature [11,35], which indicated that the aptamer successfully capture MG by specific recognition reaction. To further confirm the role of the aptamer in capturing MG, a control experiment was performed to measure the same concentration of MG using AuNPs/GQDs-WS_2_/GCE without an aptamer. After the washing step, no redox peak appeared in the cyclic voltammogram (curve b), indicating no specific adsorption of MG on AuNPs/GQDs-WS_2_/GCE surface. When aptasensor was incubated with 10 μM MG, the cyclic voltammetry response of the aptasensor in curve d remarkably increased by comparison with that in curve c, indicating that more MG molecules were captured by the aptamer to produce larger detection signal. The specific interaction between aptamer and MG can lead to MG analyte efficiently transferring from bulk solution to electrode surface, resulting in fast electron transfer between electroactive MG and aptasensor to produce strongly enhanced electrochemical signal [34]. Therefore, in combination with the specific binding of MG with aptamer and the electrocatalytic oxidation of MG by the AuNPs/GQDs-WS_2_ nanohybrids for signal amplification, a sensitive and selective electrochemical aptasensing method was proposed to determine MG based on a label-free signal amplification strategy.

In order to farther verify the role of GQDs-WS_2_ hybrid in amplification strategy, control experiments were conducted. As shown in Figure S2, CV recorded for the MCH/aptamer/AuNPs/GCE incubated with 1.0 μM MG in pH 7.4 phosphate buffer displayed a weak oxidation peak appeared at approximately +0.53 V (curve a), which indicated that MG was oxidated to produce electrochemical signal after captured onto the aptasensor surface by the aptamer recognition reaction. In contrast, the anodic peak current of MG on the MCH/aptamer/AuNPs/WS_2_/GCE (curve b) and the MCH/aptamer /AuNPs/GQDs/GCE (curve c) showed 2.1 and 2.3 times than that of in curve a, respectively, suggesting that sole WS_2_ and GQDs in the hybrid film had the ability to enhance current responses of MG. In great contrast, the largest oxidation peak of MG was observed at the MCH/aptamer/AuNPs/GQDs-WS_2_/GCE (curve d), which are approximately four times larger than that at MCH/aptamer/AuNPs/GCE (curve a). The above experiment results demonstrated that the peak current related to the electrochemical oxidation of MG captured by aptamer on the biosensor surface reduced while GQDs, WS_2_, or both are excluded to modify GCE, indicating that both WS_2_ and GQDs in the GQDs-WS_2_ hybrid film had great electrochemical activity, which can enhance the oxidation reaction of MG via electrocatalysis. Therefore, as-prepared GQDs-WS_2_ hybrids exhibited significantly enhanced electrocatalytic properties and the ability to increase current response of MG, which played an important role in promoting the oxidation of MG on the electrode surface to achieve signal amplification for sensitive electrochemical detection of MG.

### 3.4. Optimization of Experimental Conditions for Electrochemical Detection

In order to achieve better electrochemical response and high detection sensitivity, several experimental parameters were optimized, including modifier ingredient, pH, aptamer concentration, and binding time of MG. The respective figures and data are given in the Supporting Materials. The optimized conditions were (a) modifier ingredient: the composite of 1.0 mg GQDs and 1.0 mg WS_2_ dispersed in 2.0 mL doubly distilled water; (b) pH: 7.4; (c); aptamer concentration: 5.0 μM; and (d) binding time of MG: 120 min at room temperature.

### 3.5. Analytical Performance

**Figure 5:**
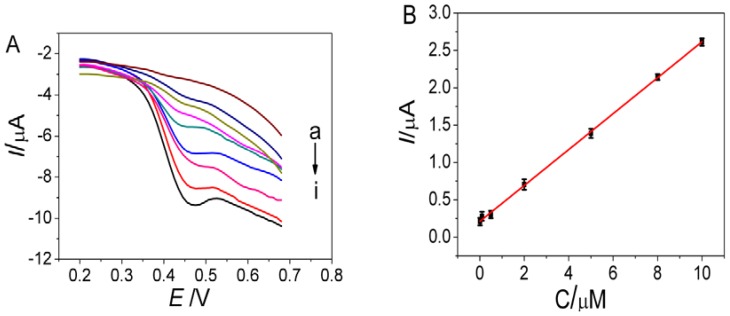
(**A**) Differential pulse voltammetric (DPV) response of the aptasensor incubated with different concentrations of MG: (**a**): 0 (**b**): 0.01, (**c**): 0.05, (**d**): 0.1, (**e**): 0.5, (**f**): 1.0, (**g**): 5.0, (**h**): 8.0, and (**i**): 10 μM. (**B**) Calibration curve corresponding to the peak current as a function of MG concentration.

Differential pulse voltammetry (DPV) was used for detection of MG due to its high sensitivity and good resolution. After incubation of the aptasensor in different concentrations of MG, the DPV signals of the aptasensor were recorded in phosphate buffer under the optimized experimental conditions. Figure 5 shows that the oxidation peak current increased with increasing the MG concentration, which was proportional to MG concentration ranging from 0.01 to 10 μM. The regression equation was I (μA) = 0.2111 + 0.2404c (μM), with a correlation coefficient of 0.9989. The detection limit (S/N = 3) was 3.38 nM for MG detection, which was much lower than many conventional electroanalytical methods reported previously (Table 1), owing to the sensitive signal amplification effect of AuNPs/GQDs-WS_2_ nanocomposite as well as the high binding affinity of aptamer to MG. Although electrochemiluminescence (ECL) method for MG detection reported in the literature [36] showed higher sensitivity with lower detection limit compared to the proposed electrochemical aptasensor; this ECL technique suffers from some undesirable limitation such as expensive instrument, operational complexity, and high cost. Furthermore, this ECL aptasensing method requires labeling for the aptamer with cyanine dye to achieve highly sensitive detection. Such a labeling process would not only make experiments relatively complex and expensive, but also affect the binding affinity between the targets and their aptamers. Qu et al. [37] developed an electrochemical sensor based on conductive carbon black paste electrode for MG determination with a detection limit of 6 nM, which had higher detection limit compared with the proposed electrochemical aptasensor with a detection limit of 3.38 nM. Moreover, some organic molecules, such as ascorbic acid, dopamine, and methylene blue, make great interference on the detection of MG due to competitive adsorption or interaction, indicating its limited selectivity for MG detection attributed to absence of MG aptamer for specific recognition to MG. In stark contrast, the proposed label-free electrochemical aptasensor on the basis of direct electrocatalytic oxidation of MG captured by aptamer avoids complicated labeling process during the sensor construction, which provided a fast, simple, and selective voltammetric method to detect MG at trace levels.

### 3.6. Selectivity, Reproducibility, and Stability

To evaluate the selectivity of the aptasensor, the developed aptasensor was used to determine MG coexisting with various potentially interfering species, such as leuco malachite green (LMG), methylene blue (MB), chloramphenicol (CAP), kanamycin (KAN) gentamicin sulfate (GEN), ascorbic acid (AA), dopamine (DA), tetracycline (TC), streptomycin (ST), and ciprofloxacin (CF). The aptasensor (MCH/aptamer/AuNPs/GQDs-WS_2_/GCE) was incubated in pH 7.0 phosphate buffer containing 5.0 μM of MG and 100 μM of interferent for 2 h at room temperature, and then it was applied to electrochemical measurement in pH 7.4 phosphate buffer using DPV. Table S1 displayed the current response of the aptasensor towards MG in the absence and presence of interference. It can be seen that current change was less than 5% in the presence of other interferent species, demonstrating that the designed aptasensor had high selectivity towards MG detection benefited from the specific aptamer recognition reaction.

Five independently prepared aptasensors were fabricated under the same conditions for the detection of 5.0 μM MG to examine the reproducibility of the aptasensor. The relative standard deviation (RSD) of 3.83% was obtained, indicating that the fabricated biosensor was repeatable and had an acceptable accuracy. The stability of the aptasensor was also investigated. When the proposed sensor was stored at 4 °C for 14 days, 95% of the initial current response to 5.0 μM MG was retained, indicating good storage stability of the aptasensor.

### 3.7. Real Sample Analysis

To evaluate the feasibility of fabricated aptasensor for the determination of MG in real samples, three spiked fish samples with various MG concentrations were assayed using the aptasensor by the standard addition method. The fish extract containing MG was diluted 1:10 in phosphate buffer. After the spiked samples were incubated with the prepared aptasensor (MCH/aptamer/AuNPs/GQDs-WS_2_/GCE) for 120 min at ambient temperature, the DPV signal was recorded. As shown in Table 2, the average recoveries in the range of 98.5–102.2% with RSD of 2.5–3.9% were obtained, which implied that the fish extract matrix had no significant effect on determination accuracy. Thus, the designed aptasensor took advantage of easy operation and high sensitivity, which could be regarded as a valid alternative to conventional assays for detection of MG in aquatic food samples.

## 4. Conclusions

In this work, Au nanoparticles/graphene quantum dots-tungsten disulfide nanosheet nanocomposite was firstly prepared and used as electroactive material for construction of novel label-free electrochemical aptasensor for sensitive determination of MG. The as-prepared AuNPs/GQDs-WS_2_ nanohybrids not only exhibited an excellent electrocatalytic effect on the oxidation of MG, but also offered an ideal platform for immobilizing aptamer molecules to fabricate biosensor. The direct electrocatalytic oxidation of MG captured by the aptamer recognition reaction avoids complicated labeling process, making operation simplified and analytical cost decreased. Due to the advantages of highly specific aptamer recognition and the WS_2_-based nanocomposite material, the aptasensor exhibits excellent sensitivity, high selectivity, satisfactory reproducibility, and good stability in the voltammetric measurement of MG. This electrochemical aptasensing platform could afford an attractive approach to MG residue analysis in aquatic samples and toxic substance analysis in the environmental contaminant monitoring application, which has a promising prospect for food safety detection and environmental analysis in the future.

## Figures and Tables

**Table 1:** Comparison of this method with other reported methods for MG determination.

+	Linear Range (M)	Detection Limit (M)	Reference
Boron-doped diamond electrode	1.0 × 10^−6^–1.0 × 10^−4^	5.0 × 10^−8^	[9]
Silica/Nafion/GCE	1.0 × 10^−6^– 6.0 × 10^−6^	3.6 × 10^−7^	[10]
Carbon nanohorns modified GCE	1.96 × 10^−7^–1.46 × 10^−4^	8.19 × 10^−8^	[11]
CeO_2_/Nafion/GCE	1.0 × 10^−6^–1.0 × 10^−5^	1.02 × 10^−6^	[12]
(GQDs/Au)_n_/GCE	4.0 × 10^−7^–1.0 × 10^−5^	1.0 × 10^−7^	[38]
Molecularly imprinted sensor	1.0 × 10^−7^–2.0 × 10^−5^	5.9 × 10^−8^	[39]
Electrochemiluminescence aptasensor	1.0 × 10^−10^–1.0 × 10^−7^	3.0 × 10^−11^	[36]
Conductive carbon black paste electrode	1.0 × 10^−8^–5.1 × 10^−7^	6.0 × 10^−9^	[37]
Carbon paste electrode	8.0 × 10^−9^–5.0 × 10^−7^	4.0 × 10^−9^	[40]
Multiwall carbon nanotube /GCE	5.0 × 10^−8^–8.0 × 10^−6^	6.0 × 10^−9^	[41]
Electrochemical aptasensor	1.0 × 10^−8^–1.0 × 10^−5^	3.38 × 10^−9^	This work

**Table 2:** Determination of MG in spiked fish samples (n = 6).

Sample	Added (µg/kg)	Found (µg/kg)	Recovery (%)	RSD (%)
1	3.65	3.73	102.2	3.9
2	7.30	7.19	98.5	3.3
3	36.50	36.35	99.6	2.5

## Supplementary Materials

The following are available online at http://www.mdpi.com/2079-4991/9/2/229/s1, Figure S1: The magnified Nyquist plot of AuNPs/GQDs-WS_2_/GCE near the origin. Figure S2: Cyclic voltammograms of (a) MCH/aptamer/AuNPs/GCE, (b) MCH/aptamer/AuNPs/WS_2_/GCE, (c) MCH/aptamer/AuNPs/GQDs/GCE and (d) MCH/aptamer/AuNPs/GQDs-WS_2_/GCE towards 1.0 μM MG in pH 7.4 phosphate buffer. Scan rate: 100 mV s^−1^.Figure S3: Effects of pH (**A**), aptamer concentration (**B**), and binding time of MG (**C**) on electrochemical responses of the aptasensor towards 5.0 μM MG in phosphate buffer. Table S1: The interference effects on MG detection.
