# Supplementary material for: Label-Free Electrochemical Aptasensor for Sensitive Detection of Malachite Green Based on Au Nanoparticle/Graphene Quantum Dots/Tungsten Disulfide Nanocomposites
Source: Nanomaterials (Basel). 2019 Feb 8;9(2):229. doi: 10.3390/nano9020229 (PMC6409966; doi:10.3390/nano9020229)
Supplement: Supplementary file 1 [file nanomaterials-09-00229-s001.pdf]

Supplementary Materials

Label-free Electrochemical Aptasensor for Sensitive Detection of Malachite Green Based on Au Nanoparticle/Graphene Quantum Dots/Tungsten Disulfide Nanocomposites

Qianqian Wang, Xiaofei Qin, Liping Geng and Yan Wang *

College of Chemistry, Chemical Engineering and Materials Science, Collaborative Innovation Center of Functionalized Probes for Chemical Imaging in Universities of Shandong, Key Laboratory of Molecular and Nano Probes, Ministry of Education, Shandong Provincial Key Laboratory of Clean Production of Fine Chemicals, Shandong Normal University, Jinan 250014, China; sdnuwqq@163.com (Q.W.); sdnuqxf@163.com (X.Q.); sdnuglp@163.com (L.G.)

* Correspondence: fagong@sdu.edu.cn; Tel.: +86-531-89212269

1. Optimization of Method

1.1. Effect of Modifier Ingredient

As the composition of electrode modifier has significant effects on the electrochemical performance of the prepared sensor, the effect of ingredient of GQDs-WS_2 modifier was studied. The mass ratios of GQDs-WS_2 were set at 1:1, 1:2 and 2:1 while the mass amount of GQDs-WS_2 keeps 2.0 mg and dispersing agent keeps 2.0 mL. The voltammetric responses of the different GQDs-WS_2 modified electrodes were examined in phosphate buffer (pH 7.4) containing 0.5 mM MG at the same operation conditions. The results of CV studies showed that the optimum proportion of the GQDs to WS_2 was 1:1. So, the composite of 1.0 mg GQDs and 1.0 mg WS_2 dispersed in 2.0 mL doubly distilled water was chosen to prepare self-assembled GQDs-WS_2 nanocomposite. Furthermore, the optimized volume of GQDs-WS_2 suspension was chosen as 5.0 μL to modify the electrode.

1.2. Effect of pH

In order to ensure the best performance of the aptamer sensor, the influence of pH of the electrolyte solution was investigated. The electrochemical responses of 5.0 μM MG at the MCH/aptamer/AuNPs/GQDs-WS_2/GCE were measured in phosphate buffer with different pH by DPV. As shown in Fig. S1A, the anodic peak current of MG increases with increasing pH values to pH 7.4, and then decreases with further increasing in the pH. Therefore, phosphate buffer with pH 7.4 was selected as supporting electrolyte for obtaining best sensitivity in all subsequent electrochemical determinations. It was also found that the oxidation peak potential of MG did not significantly shift with increasing the pH, which revealed that no proton involved in the oxidation reaction of MG.

1.3. Influence of Aptamer Concentration

The concentration of aptamer is a vital influence factor for the sensitivity of the aptasensor. Fig. S1B displayed typical current responses of different concentrations aptamer modified electrode incubating with MG. There was a gradual increase in the peak current with increasing the aptamer concentration from 1.0 to 5.0 μM, indicating that higher concentration of aptamer immobilized on the electrode surface could capture more MG molecules. However, the current response decreased slowly when the aptamer concentration exceeded 5.0 μM, which could be attributed to increased steric hindrance generated by excessive aptamer that impeded the transfer of electrons. Therefore, 5.0 μM was chosen to be the optimized concentration of the aptamer for fabrication of the aptasensor.

1.4. Influence of Binding Time

The binding time of MG with its aptamer is another important factor that affected the fabrication of the aptasensor. As displayed in Figure S1C, the current response of the aptasensor increased with the increase of binding time from 30 to 120 min and then reached a stationary plateau region at 120 min. Thus, 120 min was selected as the optimal binding time between the sensing interfaces with MG molecules in this research.

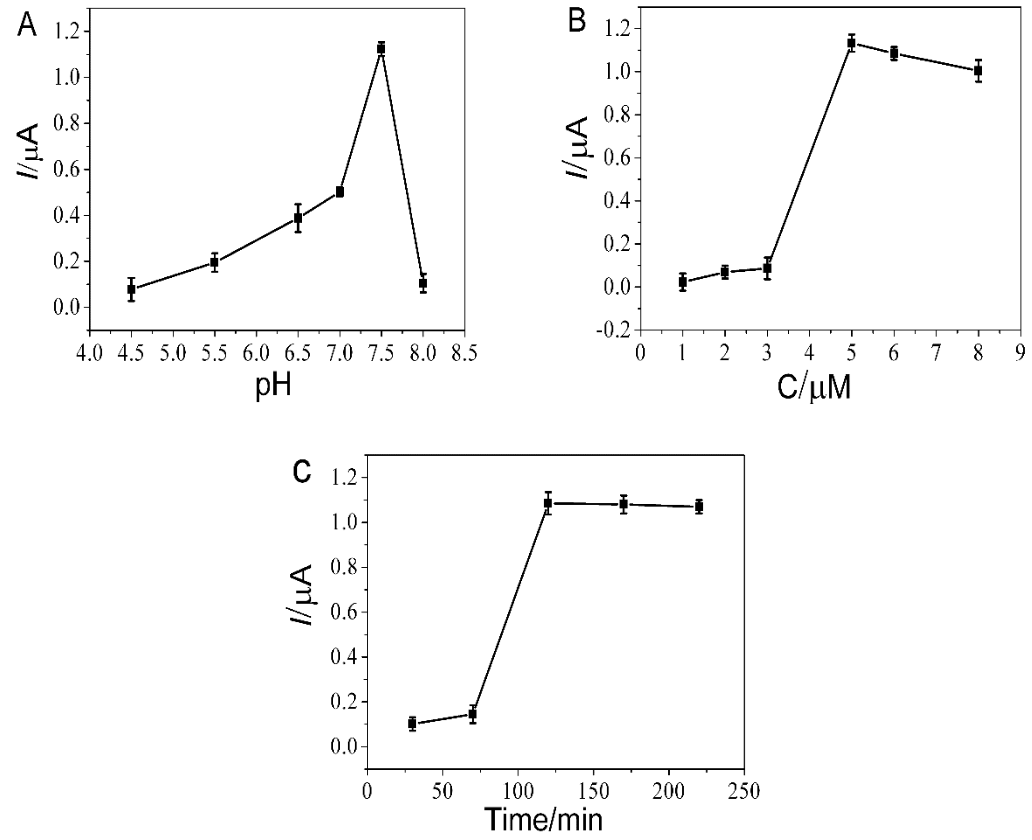

Figure S1. Effects of pH (**A**) aptamer concentration (**B**) and binding time of MG (**C**) on electrochemical responses of the aptasensor towards 5.0 μM MG in phosphate buffer.

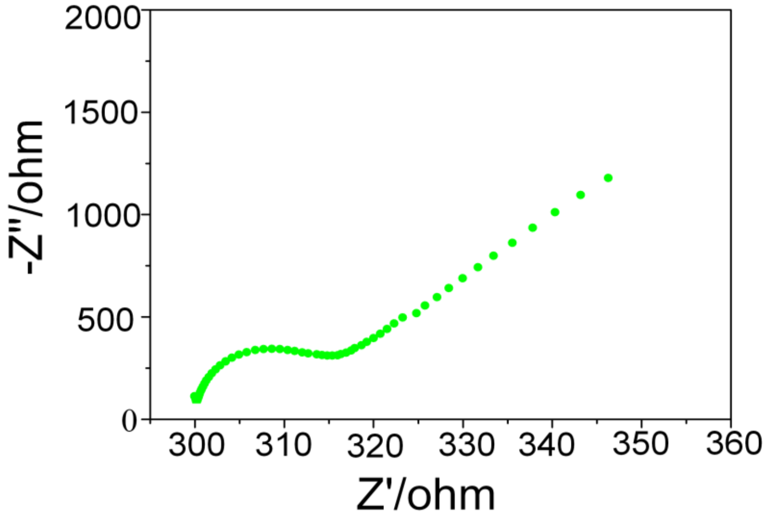

Figure S2. The magnified Nyquist plot of AuNPs/GQDs-WS_2/GCE near the origin.

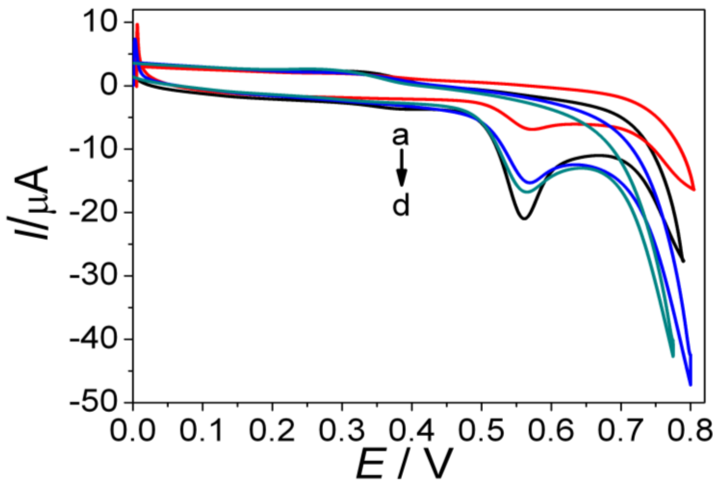

Figure S3. Cyclic voltammograms of (a) MCH/aptamer/AuNPs/GCE, (b) MCH/aptamer/AuNPs/WS_2/GCE, (c) MCH/aptamer/AuNPs/GQDs/GCE and (d) MCH/aptamer/AuNPs/GQDs-WS_2/GCE towards 1.0 μM MG in pH 7.4 phosphate buffer. Scan rate: 100 mV s^{-1}.

Table S1. The interference effects on MG detection.

Chemicals	I_p (μA)	I_p difference (μA)	Change (%)
MG	1.389	0	0
MG + LMG	1.329	−0.060	−4.31
MG + MB	1.340	−0.049	−3.52
MG + CAP	1.370	−0.019	−1.36
MG + KAN	1.373	−0.016	−1.15
MG + GEN	1.376	−0.013	−0.93
MG + AA	1.372	−0.017	−1.22
MG + DA	1.375	−0.014	−1.01
MG + TC	1.380	−0.009	−0.65
MG + ST	1.374	−0.015	−1.08
MG + CF	1.367	−0.022	−1.58
